# Liver tumor segmentation method combining multi-axis attention and conditional generative adversarial networks

**DOI:** 10.1371/journal.pone.0312105

**Published:** 2024-12-03

**Authors:** Jiahao Liao, Hongyuan Wang, Hanjie Gu, Yinghui Cai

**Affiliations:** 1 School of Computing and Artificial Intelligence, Changzhou University, Changzhou, China; 2 College of Information Science and Technology, Zhejiang Shuren University, Hangzhou, China; Shanghai Maritime University, CHINA

## Abstract

In modern medical imaging-assisted therapies, manual annotation is commonly employed for liver and tumor segmentation in abdominal CT images. However, this approach suffers from low efficiency and poor accuracy. With the development of deep learning, automatic liver tumor segmentation algorithms based on neural networks have emerged, for the improvement of the work efficiency. However, existing liver tumor segmentation algorithms still have several limitations: (1) they often encounter the common issue of class imbalance in liver tumor segmentation tasks, where the tumor region is significantly smaller than the normal tissue region, causing models to predict more negative samples and neglect the tumor region; (2) they fail to adequately consider feature fusion between global contexts, leading to the loss of crucial information; (3) they exhibit weak perception of local details such as fuzzy boundaries, irregular shapes, and small lesions, thereby failing to capture important features. To address these issues, we propose a Multi-Axis Attention Conditional Generative Adversarial Network, referred to as MA-cGAN. Firstly, we propose the Multi-Axis attention mechanism (MA) that projects three-dimensional CT images along different axes to extract two-dimensional features. The features from different axes are then fused by using learnable factors to capture key information from different directions. Secondly, the MA is incorporated into a U-shaped segmentation network as the generator to enhance its ability to extract detailed features. Thirdly, a conditional generative adversarial network is built by combining a discriminator and a generator to enhance the stability and accuracy of the generator’s segmentation results. The MA-cGAN was trained and tested on the LiTS public dataset for the liver and tumor segmentation challenge. Experimental results show that MA-cGAN improves the Dice coefficient, Hausdorff distance, average surface distance, and other metrics compared to the state-of-the-art segmentation models. The segmented liver and tumor models have clear edges, fewer false positive regions, and are closer to the true labels, which plays an active role in medical adjuvant therapy. The source code with our proposed model are available at *https*:*//github*.*com/jhliao0525/MA-cGAN*.*git*.

## Introduction

The liver is one of the most important organs in the human body for metabolism, detoxification and energy storage. However, liver cancer has become a significant disease that poses a serious threat to human health [1]. According to cancer statistics in China, the incidence of liver cancer was 9% in 2022, with a mortality rate of 12.8% [2]. As liver cancer changes, the morphological characteristics of tumors, including contour, size, and smoothness will also change. Therefore, it is crucial to segment the liver and tumor models independently from abdominal CT images, as it has significant clinical implications for disease diagnosis and liver transplantation in advanced liver cancer. In clinical practice, manual annotation is commonly used for liver and tumor segmentation, with each sample requiring more than 30 minutes [3], which is time-consuming. Moreover, due to the presence of numerous surrounding organs and tissues, some images may be obscured, resulting in insufficient clarity and deviations between the segmented tumor models and the actual conditions. This inability to effectively support assistance in medical diagnosis may even lead to misdiagnosis by physicians.

Therefore, to improve the accuracy and efficiency of diagnosis and better support clinical medical decision-making, researchers have proposed various segmentation methods. Such as Otsu’s threshold segmentation [4,5], region-growing segmentation [6], and watershed algorithm [7]. These methods mainly scan the pixels in the image and classify similar pixels based on information such as grayscale, brightness, and texture. However, these semi-automatic segmentation methods require human intervention in parameter design and adjustment. Even slight differences in parameter design can significantly impact the segmentation results, resulting in poor stability of the segmentation result and the inability to accurately extract key information.

With the emergence of machine learning and Convolutional Neural Networks (CNNs), automatic segmentation algorithms based on these techniques have been widely applied in the field of medical image segmentation [8,9]. Currently, many common automatic segmentation networks adopt the U-Net architecture, originally proposed by Ronneberger et al. [10]. Through its symmetric encoder-decoder structure and skip connections, the U-Net architecture achieves effective localization of fine-grained structures in 3D medical images. However, the U-Net still has some limitations in handling class imbalance and processing subtle structures. Researchers have made various extensions and modifications to the U-Net framework to improve its performance, such as attention mechanisms and residual connections [11,12].

In the field of Natural Language Processing (NLP), Transformer-based models [13,14] have achieved state-of-the-art performance in various tasks. The self-attention mechanism in Transformers enables the models to dynamically emphasize important features in word sequences. To further improve the accuracy and efficiency of medical image segmentation, researchers have introduced Transformer models into the segmentation pipeline. Compared to CNNs, Transformers demonstrate superior capabilities in capturing long-range dependencies and modeling complex spatial relationships [15]. By applying the Transformer architecture to the medical imaging domain, researchers have achieved significant advancements in 3D medical image segmentation tasks, enhancing segmentation accuracy [16–29].

Although Transformers excel at capturing global context and long-range dependencies, they may have limitations in generating high-quality details and textures, especially when handling intricate images. Generative Adversarial Networks (GANs), proposed by Ian Goodfellow et al. [30] in 2014, are a type of deep learning model. This model introduces a novel framework for generative modeling, aiming to generate high-quality samples through the competition of two neural networks—the generator and the discriminator. Unlike Transformers, GANs are often more efficient in generating realistic segmentation results and can perform well with limited annotated data, making them particularly advantageous in scenarios where data is scarce or computational resources are limited [31,32].

Although previous methods have made certain achievements in the field of medical image segmentation, there are still some limitations: (1) CNN-based models often employ local sliding windows or fixed receptive fields for convolutional operations, which makes it challenging to effectively model the global contextual information of the image. This can lead to poor performance of the model in medical segmentation tasks that rely heavily on large-scale contextual dependencies. (2) Transformer-based models are relatively weak in modeling spatial relationships in images and require more computational resources and memory to handle large-scale medical image data. This increases the computational cost and implementation difficulty of the algorithm. (3) The training process of GAN-based models is relatively unstable and may suffer from issues such as mode collapse or mode overfitting [33]. Additionally, the generated results of GANs may exhibit artifacts or loss of fine details.

We propose a novel multi-axis attention conditional generative adversarial network called MA-cGAN to address existing network limitations and challenges. The proposed MA-cGAN comprises a generator and a discriminator, which are trained in an adversarial manner to improve the quality of generated labels. The generator is based on a U-shaped segmentation network, and the concept of two-dimensional slice projection features (FP) is introduced. FP projects three-dimensional medical images onto different axes, extracting two-dimensional feature information from axial, sagittal, and coronal planes, thereby increasing the proportion of target segmentation content information from *O*(1/n^3^) to *O*(1/n^2^). We employ a multi-axis attention mechanism (MA) to fuse the extracted 2D features with the input features, while adaptively adjusting the weights between different axis features using learnable factors to capture key features from multiple orientations of three-dimensional medical images. The discriminator, a three-layer convolutional network, aims to accurately distinguish between labels generated by the generator and real data. To enhance the stability of the generator’s output, we utilize CT images as conditional information and concatenate them with real or fake samples as input to the discriminator, allowing for more precise control over the features and content of the generated samples.

Compared with existing segmentation algorithms, MA-cGAN combines the concepts of attention mechanisms and generative adversarial networks (GANs). It exploits attention mechanisms to capture crucial features, enabling better focus on critical regions. By makes use of GANs, MA-cGAN generates realistic segmentation results and reduces erroneous segmentation in irrelevant areas. This has significant practical implications for medical image analysis and disease diagnosis, providing doctors and researchers with more accurate and reliable tools.

In summary, this paper proposes MA-cGAN, which offers several innovations in the field of medical image segmentation:

① We propose a multi-axis attention mechanism (MA) that captures 2D feature information from different axial planes of three-dimensional medical images and fuses it with the input features, thereby enhancing the focus on crucial information.
② We constructed a U-shaped segmentation network as the generator and introduced MA. The generator generates fake labels that closely resemble the real labels based on input CT images, aiming to deceive the discriminator into misclassifying them as real labels. We also constructed a discriminator that undergoes multiple alternating adversarial training with the generator, continuously improving the quality of the generated labels.
③ We incorporated Tversky Loss and Wasserstein distance into the loss function of the GANs to effectively address class imbalance issues and alleviate the problem of mode collapse in GANs. This integration aims to enhance the quality and accuracy of segmentation results. Additionally, the optimized loss function also contributes to a better balance between the generator and discriminator during their adversarial training.
④ After data preprocessing and post-processing, MA-cGAN achieved excellent segmentation results on the LiTS test dataset. The Dice coefficient for liver segmentation reached 96.95, and the Dice coefficient for liver tumor segmentation reached 78.53. These results demonstrate the superior performance of MA-cGAN in medical image segmentation and its potential application in medical-assisted treatments.

## Related work

### CNN-based medical image segmentation

In the domain of medical image segmentation, algorithms based on Convolutional Neural Networks (CNNs) have made considerabl progress. U-Net [10] is a classic CNN-based image segmentation architecture, which consists of an encoder and a decoder, forming a symmetrical U-shaped structure. Skip connections with channel concatenation are employed between the encoder and decoder to fuse shallow and deep features. However, U-Net can only extract feature information from each individual slice, while ignoring the inter-slice feature correlations and resulting in low smoothness of the segmentation results. To overcome this limitation, Özgün Çiçek et al. [34] proposed 3D-Unet in 2016, which can directly integrate multiple slices into a 3D image for training, which improves improving the continuity of variations between adjacent image masks. Residual U-Net [12] incorporates residual connections to facilitate information flow and gradient propagation. Han et al. introduced FCN [35], a fully convolutional network that enables dense pixel-wise prediction of input images. It incorporates skip connections to fuse features at different scales, which can strengthen the model’s ability to perceive objects of different scales and capture fine details, thereby improving segmentation accuracy. While these CNN-based networks have achieved remarkable success in image segmentation, they primarily focus on local perception and feature extraction and share parameters between each convolutional layer, which may limit their capability to capture long-range contextual information. For certain medical image segmentation tasks, global context information is vital for accurate segmentation decisions, and CNNs may not fully capture these global dependencies.

### Attention mechanism for medical imaging

The attention mechanism has also been extensively applied and researched in the field of image segmentation, leading to significant advancements. O. Oktay et al. [11] proposed AttnUnet, which introduced attention mechanisms into the skip connections of U-Net. By dynamically adjusting the weights of feature maps in the skip connections, the network focuses more on crucial information, thereby enhancing segmentation accuracy. In 2017, the Transformer model [13] emerged, utilizing multi-head self-attention mechanisms to capture global contextual information more precisely. Subsequently, Dosovitskiy et al. [16] introduced Transformer to the image processing domain and proposed Vision Transformer (ViT). ViT divides the input image into multiple pixel patches and flattens them into one-dimensional sequences, effectively leveraging global image features. This approach transforms image segmentation tasks into one-dimensional sequence prediction tasks. However, compared to Convolutional Neural Networks (CNNs), Transformers perform poorly in extracting detailed features and are prone to losing fine-grained content in liver segmentation tasks. As a consequence, researchers have begun exploring the combination of CNNs and Transformers for medical image segmentation tasks.

Chen et al. [17] proposed TransUnet, which concatenates multiple Transformer encoder layers and connects them with the CNN layers in the U-Net encoder to retain the global contextual perception of Transformers and the local contextual perception of CNNs. Gao et al. [18] introduced UTNet, which extracts partial features using convolutional layers and incorporates a novel self-attention mechanism to reduce network complexity. Wang et al. [19] proposed TransBTS, incorporating four layers of ViT’s Transformer modules in the network to fuse contextual features and perform classification output through CNN modules. However, this approach increases the computational burden while enhancing segmentation accuracy. Unetr [20] removes the CNN layers in the encoder and adopts 12 Transformer encoder layers to learn the sequential representation of input volumes, effectively capturing global-scale information. Liu et al. [21] introduced Swin Transformer, which utilizes hierarchical attention mechanisms and cross-window attention mechanisms to enable Transformers to be more applicable in image processing. Subsequently, Tang et al. [22] improved the Swin Transformer module and combined it with CNNs, proposing SwinUnetr, which achieved excellent performance in 3D medical image segmentation tasks. The Transformer model models dependencies in the input sequence through self-attention mechanisms, making it highly effective in handling global information and long-range dependencies. However, when dealing with small objects that may have fewer pixels and local context, the self-attention mechanisms may not fully capture the detailed information of these small objects, leading to suboptimal performance of Transformer models in small object segmentation tasks.

### GANs in image segmentation

Traditional generative models are mainly based on probability density estimation, requiring manual specification of prior probability distributions and loss functions, and are susceptible to problems such as overfitting. Hence, Goodfellow et al. [30] proposed Generative Adversarial Networks (GANs), which enable generative models to better generate data similar to real data. To improve the stability and precision of generator output, Mirza et al. [36] introduced conditional Generative Adversarial Networks (cGANs) based on GANs. Compared to GANs that only accept noise vectors as input, cGANs introduce conditional vectors, allowing the generator to approximate the real data distribution more closely. By introducing cGANs into image segmentation tasks, the precision of segmentation results can be further improved.

P. Isola et al. [37] proposed Pix2PixGAN, which utilizes the U-Net network as the generator and incorporates the PatchGAN architecture in the discriminator. The discriminator conducts local region-based discrimination instead of whole-image discrimination, allowing it to focus more on image details and local structures, thus enhancing sensitivity to image details and achieving precise mapping from input images to target domain images. However, Pix2Pix-GAN demands a large amount of paired image data for training, i.e., matching input images with target domain images. In the domain of medical imaging, obtaining a large-scale paired dataset is arduous and costly. Beji A et al. [24] proposed a two-stage architecture, Seg2GAN. Seg2GAN generates synthetic data distributions to improve the quality of input images, and then uses the new data for segmentation. Experiments conducted on datasets such as retinal and coronary vessels show that using the new synthetic data results in better segmentation performance compared to models trained only on original real images. Guo K et al. [25] addressed issues such as model collapse, gradient vanishing, and convergence failure in GANs by proposing MedGAN, which uses Wasserstein loss as a convergence measure to assess the convergence of the generator and discriminator. The model’s advantages in terms of convergence, training speed, and the visual quality of generated samples have been validated on multiple datasets, including scabies and blisters. Additionally, GAN-based medical image segmentation algorithms may encounter problems such as mode collapse and mode oscillation, and the training process is frequently unstable, especially in the initial phases and parameter selection of the model. This may lead to difficulty in achieving convergence during the training process, requiring more time and computational resources to obtain satisfactory segmentation results.

### Transformer in generative adversarial networks

Inspired by the notions of Transformers and GANs, researchers have put forward a series of networks that combine Transformer and GANs [38–43]. Esser et al. [14] introduced a novel self-attention mechanism that takes into account both local and global contextual information to handle long-range dependencies in images, thereby better grasping the structure and details of the images. They combined it with a convolutional GANs model to learn context-rich visual feature codebooks, facilitating high-resolution image synthesis. Wang et al. [38] proposed TransGAN, which applies the Transformer architecture to image generation tasks, converting it into an unconditional GANs framework. By capturing long-range dependencies and modeling global context, TransGAN can generate high-quality medical images. However, when applied to medical image segmentation tasks, TransGAN might face the challenge of insufficient explicit guidance at the pixel-level annotation. P. Kumar et al.[26] proposed a digital artwork restoration method based on Generative Adversarial Networks (GANs) that incorporates dual (spatial and channel) attention and channel transformers. The method uses spatial and channel attention layers in the encoder part of the generator and employs channel transformers in the skip connections. This approach achieves multi-scale feature fusion and reduces the semantic gap between the encoder and decoder layers. The method outperforms existing artwork restoration techniques on two datasets. Transformers can help networks better capture global information in images, particularly for structures that cover large spatial extents in segmentation tasks. The application of GAN in medical image segmentation tasks enables the network to generate more realistic segmentation results. However, combining Transformers and GANs can result in increased computational complexity, requiring more computational resources and time for training and inference, fundamentally limiting its applicability.

## Methodology

**Fig 1:**
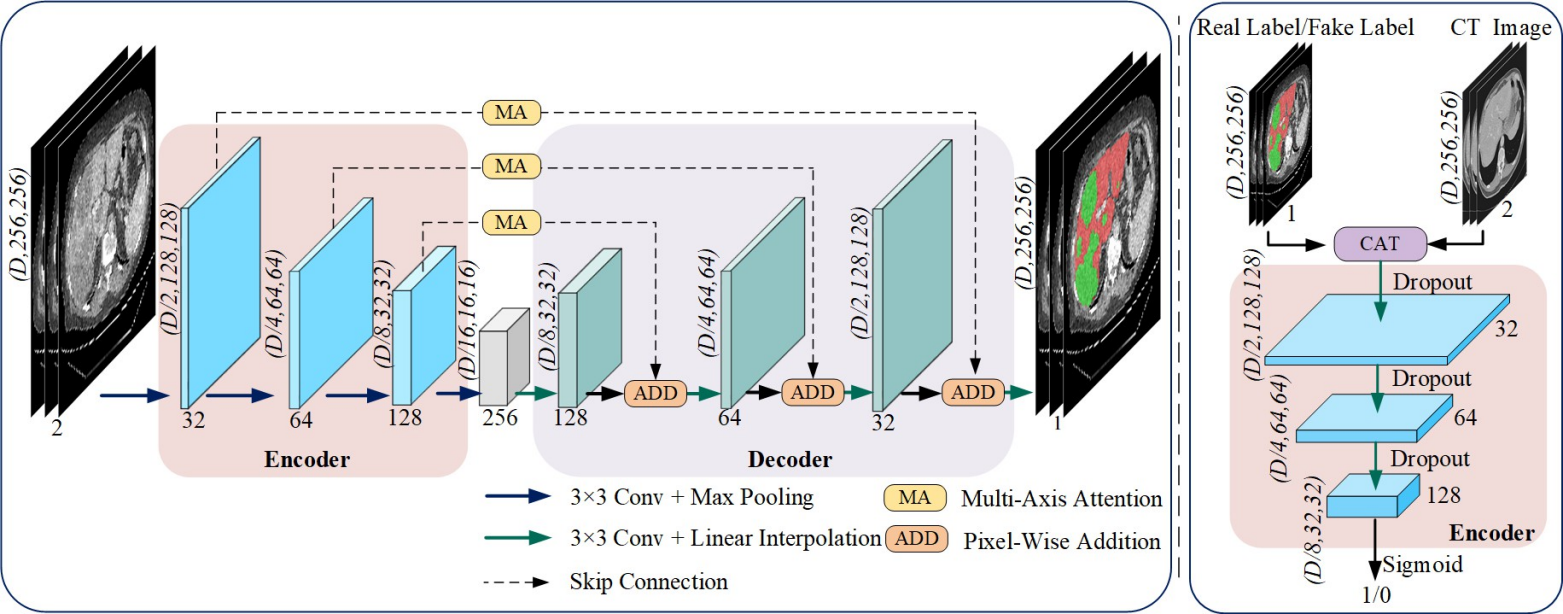
Overview of MA-cGAN. The generator consists of an encoder and a decoder, with both parts structurally symmetrical. The CT image (2×D×H×W) is input to the generator, and the generator outputs the segmentation result (1×D×H×W) after processing. The discriminator consists of only one encoder, which takes the CT image (2×D×H×W) as conditional information. The CT image and the label (1×D×H×W) are concatenated and used as input (3×D×H×W) to the discriminator. The discriminator determines the probability of the input label being a real label and outputs the corresponding result. (a)Generator (b)Discriminator.

The overall network architecture of our proposed MA-cGAN is illustrated in Fig 1. It consists of a generator and a discriminator. The generator is a U-Net-based segmentation network, composed of four parallel networks. Skip connections between these networks are established using a multi-axis attention mechanism (MA). The discriminator is a three-layer network responsible for assessing the authenticity of the input labels and providing feedback to the generator to update its parameters.

### Generator

The network architecture of the generator is a sophisticated three-dimensional segmentation network specifically designed for end-to-end segmentation of 3D medical images. As depicted in Fig 1A, the architecture features a carefully constructed encoder-decoder structure. The encoder is composed of four sequential downsampling layers that progressively contract the spatial dimensions of the input image while expanding the depth. Starting from an initial image size of C×D×256×256, where C represents the number of channels, D the depth, and 256 × 256 the spatial dimensions, the encoder reduces this to 256×D/16×16×16 through a series of convolutional and pooling operations. This process is crucial for extracting and compressing high-level semantic features from the input medical images, allowing the network to capture complex patterns and structures inherent in the data.

The decoder, in contrast, is designed with four upsampling layers that gradually expand the feature maps back to the original image dimensions. The decoder aims to reconstruct the image to match the pixel-level segmentation results with the ground truth labels. To ensure consistency, the shape of the interpolated image aligns with that of the image before pooling in the corresponding encoder layer. This process is facilitated by skip connections, which bridge the encoder and decoder layers, allowing for the transfer of fine-grained low-level features that complement the high-level features processed in the decoder.

To enhance the feature extraction capabilities, the network incorporates Multiple-Angle (MA) attention mechanisms. These mechanisms leverage information from different axes to ensure comprehensive feature capture from various perspectives. Convolutional kernels throughout the network are uniformly set to 3×3, which simplifies the model while preserving high segmentation accuracy [44]. Batch Normalization (BN) layers [45] are employed to normalize the outputs of the convolutions, which helps to accelerate the training process and improve the generalization performance of the network. Given the extensive number of parameters in the generator, the ReLU activation function is applied after both upsampling and downsampling operations. This choice of activation function is instrumental in mitigating overfitting by retaining only the positive values of the input features, thus enhancing the model’s robustness.

### Dicscriminator

We designed a discriminator network comprising three convolutional layers, as illustrated in Fig 1B, specifically to differentiate between real and synthetic input images. The network takes as input a CT image that is concatenated with a label indicating whether it is real or generated. This concatenated input is processed through three downsampling layers, which progressively reduce the spatial dimensions while increasing the depth of the feature maps. Each convolutional layer applies a series of filters to extract and learn high-level feature representations from the input image. The downsampling operations are designed to capture complex patterns and textures that are crucial for distinguishing real images from those generated by the generator.

After the convolutional operations, the extracted features are fed into a fully connected layer, which ultimately uses a sigmoid activation function to produce a probability score. This score reflects the likelihood that the input sample is real data. A score close to 1 indicates high confidence in the input being real, while a score close to 0 suggests that the input is more likely a fake sample generated by the generator. This probabilistic output is critical for guiding the training process, ensuring that the discriminator effectively challenges the generator to improve the quality of its outputs.

To maintain the balance between the discriminator and the generator and to prevent the discriminator from becoming too dominant, which could lead to training instability, a dropout layer with a rate of 0.5 is incorporated after each downsampling layer. This dropout layer randomly omits a portion of neurons during training, which helps to regularize the model and prevent overfitting. By introducing this stochastic element, the network maintains robustness and ensures that the discriminator’s learning does not overshadow the generator’s progress.

### Multi-axis attention mechanism (MA)

**Fig 2:**
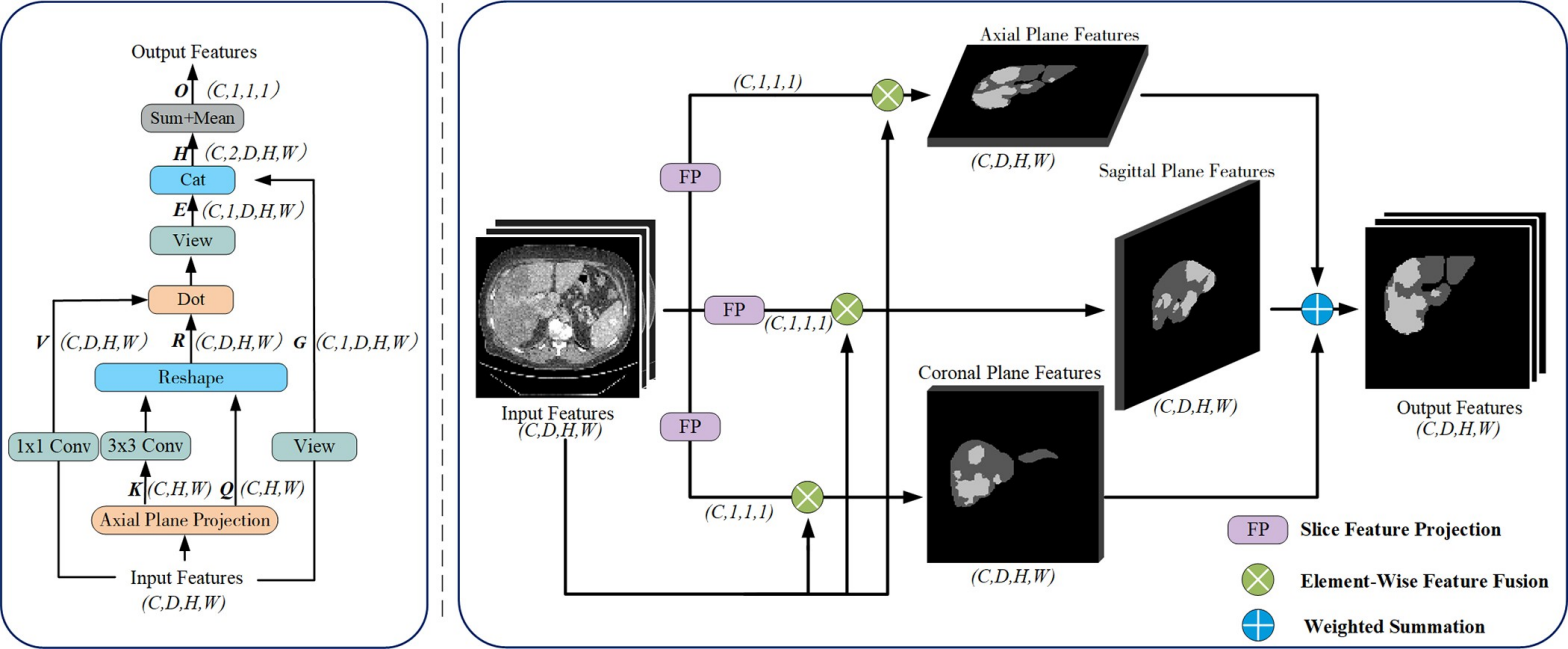
Overview of multi-axis feature extraction structure. In MA-cGAN, we first employ slice feature projection (FP) to project the 3D image feature information along axes onto a 2D plane. Then, the obtained 2D feature information from multiple directions is fused with the input features. Finally, a learnable factor is applied to weight the fused output, enabling the comprehensive integration of slice feature information from different axes. (a) Slice Feature Projection(FP) (b) Multi-Axis Attention Mechanism(MA).

The Slice Feature Projection Module (FP) is introduced in MA, which projects the 3D medical images onto a 2D plane along a specific axis, enhancing the proportion of target information. If the proportion of target segmentation content before projection is *O*(1/n^3^), after projection to the 2D plane, the proportion is improved to *O*(1/n^2^), reducing the loss of critical information. The FP structure, as illustrated in Fig 2A, obtains relevant information on the projection axes by globally average-pooling and max-pooling the input features (C×D×H×W) and then summing them. This process generates the key (*K*) and query (*Q*) values. The shapes differ when projected onto different axes planes. When projected onto the axial plane, the shape is C×H×W; when projected onto the sagittal plane, the shape is C×D×H; and when projected onto the coronal plane, the shape is C×D×W. The specific formulas are defined as shown in Eq 1.


K=Q=Add(Max(x),Mean(x)) (1)

The value tensor (*V*)is obtained by applying a 1×1 convolution to the input features, preserving its shape (C×D×H×W). The key (*K*)is processed using two 3×3 convolutions and concatenated with the query (*Q*). The tensor K is subjected to two consecutive 3x3 convolutions followed by concatenation with Q. Then, two 1x1 convolutions and one Unsqueeze operation are applied to expand the dimensions and replicate along the depth dimension, ensuring the shape remains consistent with the input features, resulting in an attention matrix *R* (C×D×H×W). The attention matrix R is then broadcast-multiplied with the value (V), and the multiplication result is concatenated with the input features after expanding its dimensions. This process generates a mixed attention matrix *H* (C×2×D×H×W). Finally, the mixed attention matrix H is summed along the depth dimension and averaged, resulting in the two-dimensional projected feature information (C×1×1×1).

Three FP modules were employed in MA, projecting the information of three-dimensional medical images onto axial, sagittal, and coronal planes respectively. This approach facilitated the acquisition of two-dimensional projection features in three different directions. The two-dimensional projected feature information is fused with the input feature through element-wise multiplication, followed by weighted summation using three learnable factors to achieve the fusion of multidimensional features, as illustrated in Fig 2B. MA effectively utilizes the multi-angle information of the input image, preserving more detailed features. The formulation for the fusion process is given by Eq 2.


$$y=\sum_{\alpha =1}^{3}\beta_{\alpha }\cdot \mathrm{FP}(\alpha )\cdot x,\sum_{\alpha =1}^{3}\beta_{\alpha }=1$$ (2)

Whereas *α* represents the three projection directions, *β*_*α*_ represents the learnable weighted factors in each direction, FP(·) represents the two-dimensional feature information obtained by projecting along a specific direction, and *x* represents the input features.

### The training process of Generative adversarial networks (GANs)

**Fig 3:**
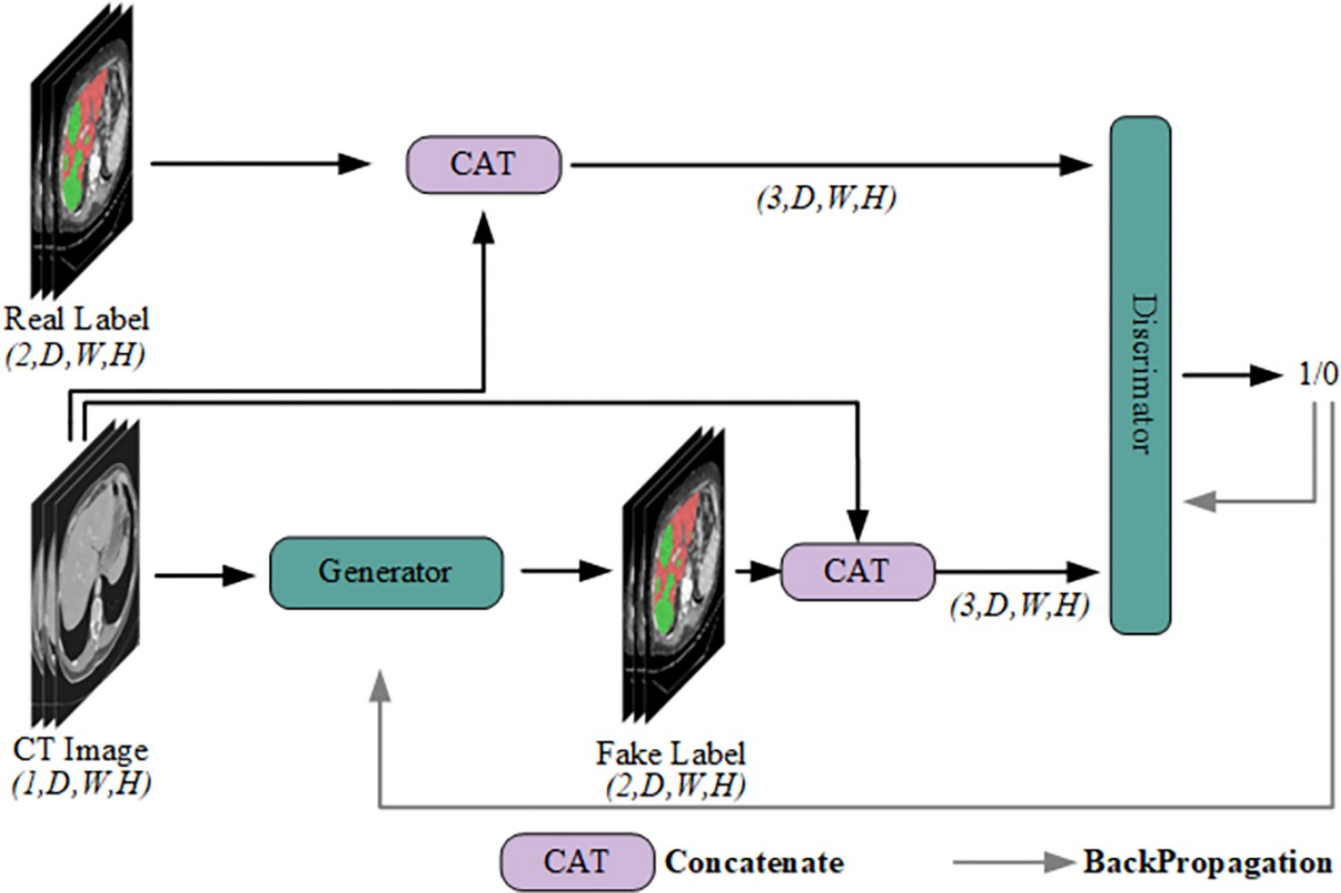
Overview of alternating adversarial training process. In the alternating adversarial training process, the generator model generates samples to deceive the discriminator model, while the discriminator model provides feedback by accurately distinguishing between the generated samples and real samples. The generator and discriminator compete and cooperate, iteratively optimizing the adversarial loss function between them until the generator can generate realistic samples.

The training process of the generative adversarial network is illustrated in Fig 3, involving iterative training between the generator and the discriminator. The generator’s task is to generate fake labels that resemble the real labels as much as possible, with the expectation that the discriminator will classify the fake labels as real. Initially, the CT image is passed through the generator segmentation network, which performs a series of operations such as downsampling, upsampling, and skip connections, to output fake labels. The CT image is concatenated with the fake labels and fed into the discriminator. The loss between the discriminator’s output and a matrix of all ones is computed, and then backpropagation is used to update the weights of the generator, enhancing its ability to generate more realistic fake labels. This completes a single training step for the generator. After completing a single training step for the generator, its parameters are frozen, and training is performed for the discriminator.

The task of the discriminator is to distinguish between real labels and fake labels generated by the generator. The CT image is used as a conditioning factor and is concatenated with either real or fake labels before being input into the discriminator. Based on the authenticity of the input labels, the loss is computed between the discriminator’s output and a matrix of all ones or all zeros. Through backpropagation, the discriminator’s weights are updated to improve its ability to discriminate between real and fake labels, completing a single training step for the discriminator.

By iteratively training the generator and the discriminator, the quality of the generated labels and the discriminative accuracy of the discriminator are continuously improved. This process continues until the generated fake labels are sufficiently close to the real labels, and the discriminator is no longer able to accurately distinguish between real and fake labels, achieving a Nash equilibrium. This marks the completion of the training process for the generative adversarial network. At this point, the generated fake labels possess high quality and can be used for high-precision liver and tumor segmentation in unlabeled CT images.

### Loss function

In traditional generative adversarial network, the loss function is defined as Eq (3):

$$\underset{G}{\min}\;\underset{D}{\max}V(D,G)=E_{x∼p_{data}(x)}[\log D(x)]+E_{z∼p_{z}(z)}[\log(1-D(G(z)))]$$ (3)

Where *E* represents the expectation value of the distribution function, *x* represents the preprocessed CT image, *z* represents the noise input to the generator, *G*(*z*) represents the generated fake image by the generator, and *D*(*x*) represents the discriminator’s probability of judging an image as real. For the discriminator *D*, the goal is to classify real data *x* as true and fake data *G*(*z*) as false, which means *D*(*x*) should approach 1 and *D*(*G*(*z*)) should approach 0. The objective is to maximize *V*(*D*,*G*). On the other hand, for the generator *G*, the objective is to have the discriminator classify fake data *G*(*z*) as real, which means *D*(*G*(*z*)) should approach 1. The objective is to minimize *V*(*D*,*G*). This optimization approach uses the JS divergence, which can result in fixed gradients and prevent updates to the generator when the two distributions have no overlap or negligible overlap. Wasserstein GAN [46] addresses this issue by using the Wasserstein distance, which is defined in Eq (4) as follows:

$$W(P,Q)=\inf_{r\in \prod (P,Q)}E(x,y)[\|x-y\|]$$ (4)

Where inf represents the maximum lower bound, ∏(*P*,*Q*) represents the joint distribution set combining distributions *P* and *Q*, and *E*(*x*,*y*) represents the expectation. Unlike JS divergence, the Wasserstein distance not only considers the differences between distributions but also takes into account the minimum cost of transforming one distribution into another. It can measure the distance between two distributions. We removed the log function from the loss function, which reduces the issues of gradient vanishing and exploding, resulting in a more stable training process.

Tversky Loss was proposed by Tversky et al. [47] in 2017 to address the issue of high precision but low recall in image segmentation caused by class imbalance. The formula for Tversky Loss is given by Eq (5):

$$T(y,G(z,y))=\frac{\sum_{i=1}^{N}y_{i}\cdot G{\left(x\right)}_{i}}{\sum_{i=1}^{N}y_{i}\cdot G{\left(x\right)}_{i}+\alpha \sum_{i=1}^{N}(1-y_{i})\cdot G{\left(x\right)}_{i}+\beta \sum_{i=1}^{N}y_{i}\cdot (1-G{\left(x\right)}_{i})}$$ (5)

Where *y*_*i*_ in *T*(*y*,*G*(*z*,*y*)) represents the i-th voxel as a lesion voxel, 1−*y*_*i*_ represents the i-th voxel as a normal voxel. *G*(*x*)_*i*_ represents the probability of predicting the i-th voxel as a lesion voxel, and 1−*G*(*x*)_*i*_ represents the probability of predicting the i-th voxel as a normal voxel. (1−*y*_*i*_)⋅*G*(*x*)_*i*_ represents the false positive case, *y*_*i*_⋅(1−*G*(*x*)_*i*_) represents the false negative case, and *α* and *β* respectively denote the parameters controlling the weight of false positive and false negative.

We incorporated additional conditional information and introduced Tversky Loss into the loss function, effectively addressing the issue of class imbalance and further enhancing the segmentation performance of the generator. The loss function is defined as Eq (6) below:

$$L(G,D)=\underset{G}{\min}\;\underset{D}{\max}V(D,G)=E_{x∼p_{data}(x)}[D(x,y)]-E_{z∼p_{z}(z)}[D(G(z,y))+T(y,G(z,y))]$$ (6)

## Experiments

### Dataset

We conducted image segmentation experiments on the LiTS dataset [48]. The dataset consists of 131 abdominal CT image files with annotations for liver and liver tumors. Each file varies in terms of liver size, shape, and pathological features. The abdominal CT images and corresponding annotations have a shape of depth × width × height (D × W × H), where both W and H are 512, and D ranges from 86 to 987. In our experiments, we excluded 21 CT images with inaccurate annotations, resulting in a total of 110 CT images. Using random sampling, we selected 80 images as the training set, 10 images as the validation set, and the remaining 20 images as the test set.

**Fig 4:**
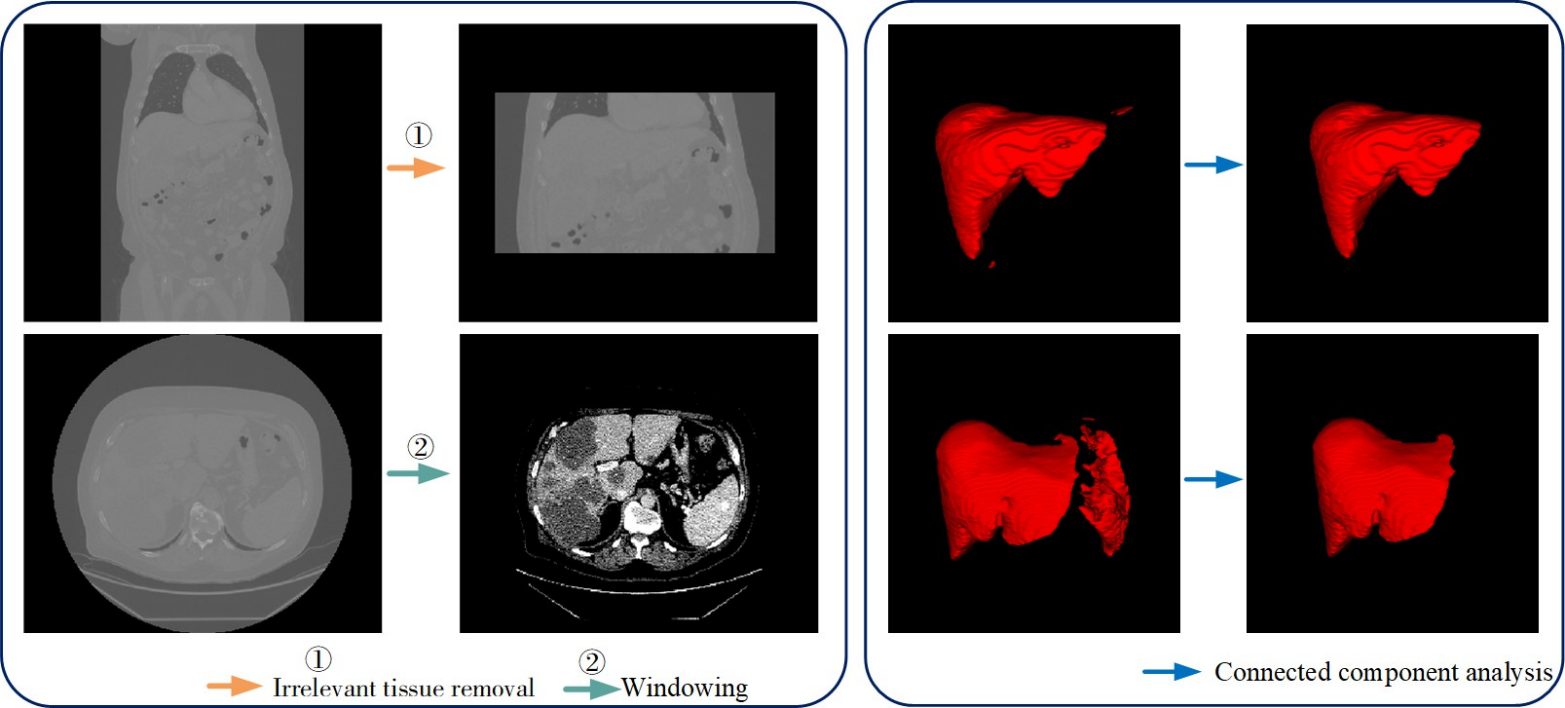
Data processing results. before segmentation. The images were subjected to windowing to enhance the contrast between the tumor and surrounding tissues, making the target region more prominent. After segmentation, a connected component analysis was performed to remove noise and undesired regions that did not conform to the expected shape, resulting in a more continuous, complete, and accurate segmentation. (a) Data pre-processing (b) Data post-processing.

To reduce the impact of irrelevant tissues in CT images on the segmentation results, we performed two preprocessing operations on the dataset: ① Irrelevant tissue removal: Along the depth direction of the slices, we identified the start and end indices of the labeled voxel points and removed the slices outside this index range, thereby reducing the interference from other abdominal organs during liver segmentation. After preprocessing, the image depth (D) values ranged from 28 to 312. ② Windowing: We set the window level of the CT images to 70 Hounsfield Units (HU) and the window width to 100 HU. This adjustment resulted in the intensities of the CT images ranging from -30 HU to 170 HU. Tissues with intensities below -30 HU were displayed as black, while those above 170 HU were displayed as white. This setting reduced the influence of excessively high or low voxel intensities on the experiment and improved the contrast of the liver and tumors in the CT images. The effects of the preprocessing are illustrated in Fig 4A. After CT image preprocessing, irrelevant slices to liver segmentation were removed to reduce interference from other abdominal organs during the liver segmentation process. Simultaneously, the grayscale display range was redefined, biasing the image towards higher density values, thus highlighting the density differences in liver tissue and further enhancing image clarity. This processing enables clearer visualization of structures with different densities and facilitates differentiation and identification of low-density abnormal tissues such as tumors, thereby improving the network’s capability for feature extraction of the liver and tumors.

There is only one liver in the human body. If there are two or more regions in the liver segmentation result, it indicates that false positive regions may be present in the segmentation results. To address this, we performed connected component analysis on the liver segmentation result, dividing it into multiple connected components. Assuming that the largest connected component has x voxels, if there are connected components with voxel counts less than rate×x in the segmentation result, they are removed. In this study, we set the rate to 0.3. The post-processing effect is illustrated in Fig 4B. After connected component analysis and voxel removal operations, significant changes were observed in the data content of the liver segmentation result. By removing small volume connected components, the influence of noise voxels was reduced, leading to a significant decrease in noise levels in the data and an enhancement of the saliency and recognizability of the liver structures. Additionally, the overall distribution of voxel values became more concentrated around liver tissue representation, reinforcing the representation of liver structures in the data and improving the accuracy of the segmentation result.

### Evaluation metrics

We evaluated the experimental results and the model using multiple metrics, including Dice score [49], 95 Hausdorff distance (HD95) [50], average surface distance (ASD) [51], and Jaccard Index(Jaccard) [52].

In medical image segmentation, the Dice coefficient is used to measure the similarity between the segmentation result and the ground truth. Let *S* represent the segmentation result and *G* represent the ground truth. The formula for the Dice coefficient is given by Eq (7):

$$d_{Dice}(S,G)=\frac{2|S\cap G|}{\left|S|+|G\right|}$$ (7)

The Dice coefficient calculated using this formula ranges from 0 to 1, where 1 indicates perfect overlap, meaning the segmentation result is identical to the ground truth, and 0 indicates no overlap. The Dice coefficient is one of the standard metrics for evaluating the performance of segmentation algorithms.

Hausdorff distance is used to measure the maximum error between the segmentation result and the ground truth. It reflects the extreme differences between the segmentation result and the ground truth, making it particularly suitable for measuring boundary accuracy. Let *S* and *G* denote the segmentation result and the ground truth, respectively. The definition of Hausdorff distance is given by Eq (8):

$$d_{H}(S,G)=\mathrm{Max}\{h(S,G),h(G,S)\};h(S,G)=\underset{s\in S}{\max}\;\underset{g\in G}{\min}\|s-g{\|}^{2}$$ (8)

To avoid unreasonable distances caused by certain points being too far from the main cluster, this study calculates the distance of the furthest 95% of pixels as a metric (HD95) to ensure numerical stability. The distance values are bounded by the 95th percentile, making the distance measurement more robust and less sensitive to outliers. A smaller HD95 indicates that the two sets are closer to each other, indicating a better segmentation result.

ASD represents the average distance between the segmented results and the ground truth edges. Compared to HD95, ASD provides a more comprehensive assessment of segmentation accuracy. A smaller ASD indicates a closer match between the segmentation results and the ground truth labels. Let *S* represent the segmentation result and *G* represent the ground truth. The formula for ASD is given by Eq (9):

$$d_{ASD}(S,G)=\frac{1}{2}(\frac{1}{n_{S}}\sum_{s\in S}d(s,G)+\frac{1}{n_{G}}\sum_{g\in G}d(g,S))$$ (9)

However, Hausdorff focuses on the distance between the most dissimilar point pairs, whereas ASD is based on the average distance between all points. Therefore, ASD can provide a more comprehensive assessment of the accuracy of segmentation results.

The Jaccard measures the overlap between the predicted and ground truth values. A larger Jaccard indicates a higher degree of overlap between the two sets, indicating a smaller difference between the segmentation results and the ground truth annotations. Let S represent the segmentation result and G represent the ground truth. The formula for the Jaccard index is given by Eq (10):

$$d_{Jaccard}(S,G)=\frac{\left|S\cap G\right|}{\left|S\cup G\right|}$$ (10)

### Implementation details

We implemented MA-cGAN on PyTorch 1.8.0. All models were trained on an RTX 3090 with 24GB of memory. We used AdamW [53] as the optimizer with an initial learning rate of 2e-4. During training, we monitored the average Dice coefficient of the liver and tumor on the validation set as the evaluation metric. If the Dice coefficient did not improve after 30 epochs, we halved the learning rate and continued training. Training was stopped either after 60 epochs without improvement or when the number of epochs reached 300. The input images were resized to a width and height resolution of 256×256, and the batch size was set to 2. After training, we visualized the experimental segmentation results using ITK-SNAP for comparison.

### Quantitative evaluations

To evaluate the segmentation performance of the MA-cGAN method, we conducted comparisons on the LiTS dataset’s test set. The comparison of segmentation results from multiple models is shown in Table 1.

**Table 1:** Quantitative segmentation results on the LiTS dataset.

Model	Liver				Tumor				Params	Flops
	Dice	HD95	ASD	Jaccard	Dice	HD95	ASD	Jaccard		
3D-Unet [34]	94.37	6.85	1.18	91.18	61.35	25.67	7.38	50.61	5.24M	678.58G
AttnUnet [11]	95.47	7.24	1.24	90.84	57.46	23.24	6.96	44.65	6.40M	573.46G
TransUnet [17]	95.62	6.25	1.04	92.65	64.81	18.90	7.19	53.81	22.29M	689.11G
UTNet [18]	96.54	2.07	**0.51**	93.84	67.95	20.09	5.97	55.96	28.10M	891.39G
TransBTS [19]	96.36	2.64	0.63	93.15	70.34	29.92	4.95	56.48	95.53M	34.73G
Unetr [20]	95.34	4.68	0.85	91.87	66.81	21.55	6.12	53.62	92.97M	193.42G
SwinUnetr [22]	96.28	3.70	0.68	92.98	76.89	29.85	5.60	61.31	4.08M	49.69G
MA-cGAN	**96.95**	**2.15**	0.54	**94.54**	**78.53**	**12.12**	**2.01**	**65.24**	4.99M	41.93G

It can be observed that the segmentation performance of the MA-cGAN network surpasses that of previously proposed networks. After data preprocessing and segmentation post-processing, the liver model achieves a Dice score of 96.95, and the tumor model achieves a Dice score of 78.53. These scores are 0.41 (UTNet [18]) and 1.64 (SwinUnetr [22]) higher than the second-best segmentation methods, respectively. MA-cGAN achieves excellent segmentation performance while utilizing a smaller parameter count. This indicates that our proposed multi-axis attention mechanism can better focus on small regions of interest and exhibit good segmentation performance on the irregular boundaries of the liver and tumors.

**Fig 5:**
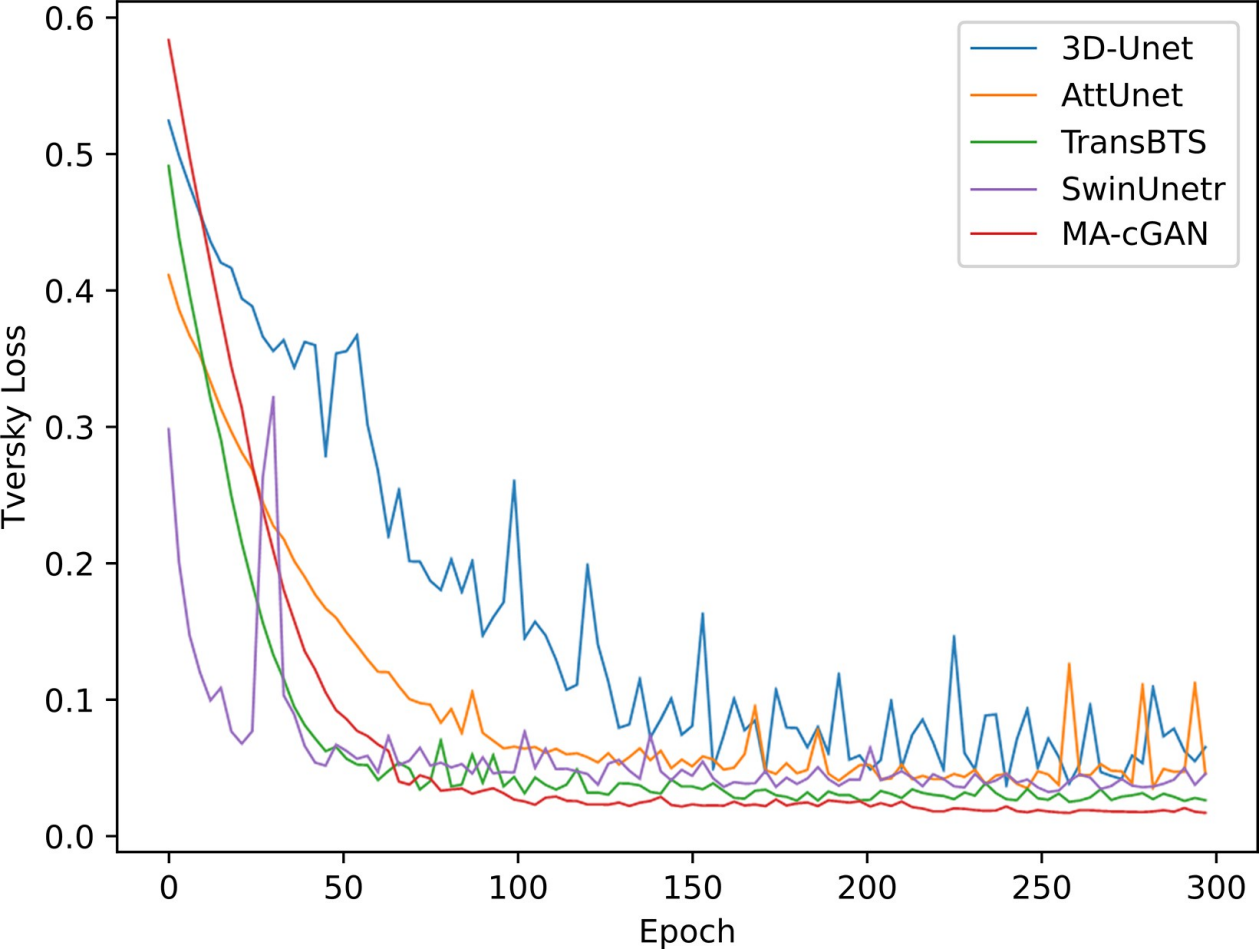
Comparison of Tversky Loss on the validation set during the training process of different models. After convergence, MA-cGAN exhibits a lower Tversky Loss, demonstrating its excellent segmentation performance for liver segmentation tasks.

We also visualized the changes in average Tversky Loss on the validation set during the training process of 3D-Unet [34], AttnUnet [11], TransBTS [19], SwinUnetr [22], and MA-cGAN. The descending curves are illustrated in Fig 5.

It can be observed that MA-cGAN exhibits a relatively slower rate of Tversky Loss reduction. This is due to the unique nature of the generative adversarial network, where the optimization objectives of the generator and discriminator are inherently conflicting. The generator aims to generate samples that resemble real data distributions, while the discriminator aims to differentiate between the generated samples and real samples. This adversarial training process makes the training of MA-cGAN more challenging compared to traditional supervised learning models. However, with increasing training iterations, the segmentation accuracy of MA-cGAN gradually improves, and its Tversky Loss becomes lower than the other compared models. This indicates that although MA-cGAN requires a longer convergence time during training, it achieves higher segmentation accuracy in medical segmentation tasks, minimizing the occurrence of false negatives and reducing the potential for diagnostic errors by medical professionals. This improvement enhances the accuracy and reliability of medical diagnosis.

**Fig 6:**
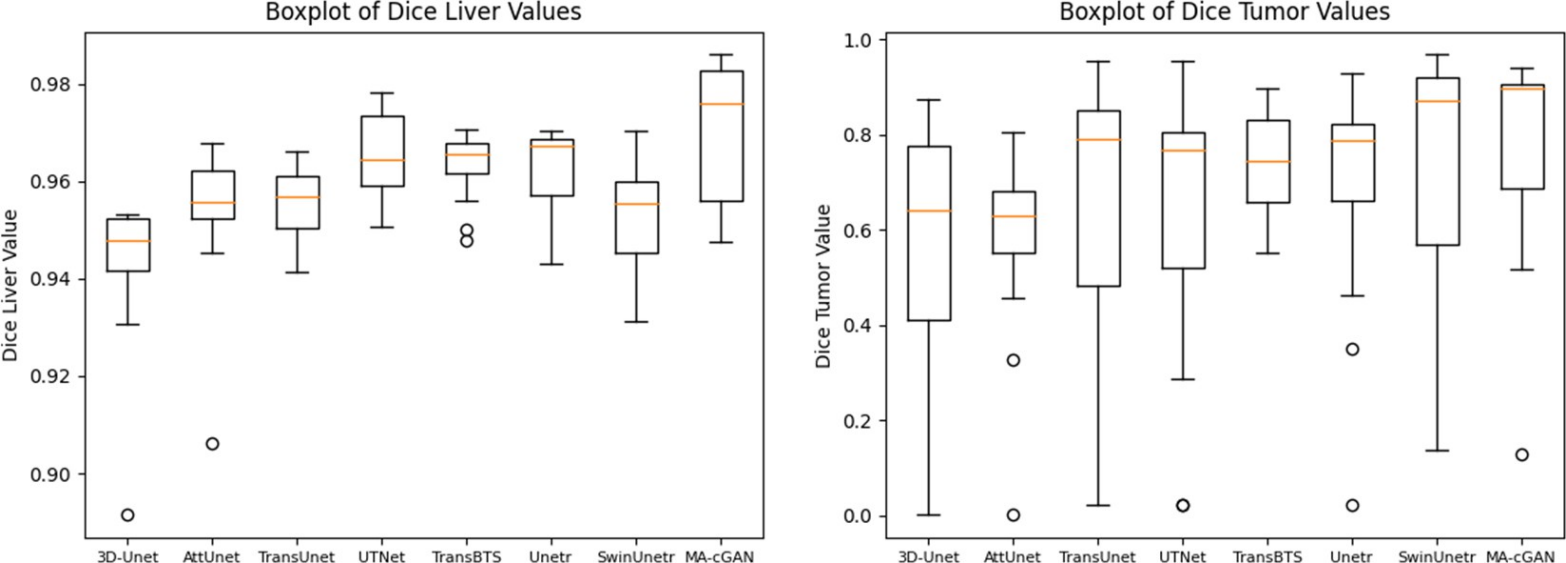
The box plots of liver and tumor segmentation results. The box plots demonstrate that MA-cGAN exhibits better segmentation accuracy and stability compared to other networks. (a) Boxplot of Dice Liver Values (b) Boxplot of Dice Tumor Values.

In order to better evaluate the performance of each model in liver and tumor segmentation, we created box plots for comparison, as shown in Fig 6A and 6B.

These two figures respectively illustrate the Dice coefficient values for different network models in liver and tumor segmentation tasks. Fig 6A shows the box plot of Dice coefficients for liver segmentation, while Fig 6B presents the box plot for tumor segmentation. The figures include performances of 3D-Unet, AttUnet, TransUnet, UTNet, TransBTS, Unetr, SwinUnetr, and our proposed MA-cGAN model.

In the liver segmentation task, the MA-cGAN model demonstrates the highest distribution of Dice coefficients, with a median close to 0.98, indicating its superior performance in liver segmentation. Additionally, the box for the MA-cGAN model is taller, suggesting a high stability and a narrow range of fluctuation in its predictions, further confirming its consistency and reliability across different samples. In contrast, other models exhibit some shortcomings. The Dice coefficient distributions for the 3D-Unet and AttUnet models are lower, with some outliers, indicating potential underperformance in certain cases. The performances of the TransUnet, UTNet, and TransBTS models are relatively similar, with means and medians around 0.95. Although these models show stable performance, they slightly lag behind MA-cGAN in terms of highest values. The Unetr and SwinUnetr models perform slightly worse than TransUnet, UTNet, and TransBTS, and the presence of outliers in the 3D-Unet and TransBTS models suggests that their prediction results may deviate from the normal range in specific situations.

In the tumor segmentation task, the MA-cGAN model again exhibits excellent performance, with a median Dice coefficient close to 0.85 and a tall box, indicating stability and consistency in tumor segmentation. In comparison, the other models show significant variability in performance. The Dice coefficient distributions for the 3D-Unet and AttUnet models are lower, with multiple outliers, highlighting instability in tumor segmentation. The performances of the TransUnet and UTNet models are relatively similar, with means and medians around 0.75. Although these models are stable, they fall short of MA-cGAN in terms of the highest values. The TransBTS, Unetr, and SwinUnetr models perform slightly worse than TransUnet and UTNet, with multiple outliers, indicating that their predictions may deviate from the normal range in certain situations.

In summary, the MA-cGAN model demonstrates outstanding performance in both liver and tumor segmentation tasks, achieving the highest Dice coefficients and the smallest range of fluctuations, proving the superiority of our proposed network in these tasks. Although other models also perform well, they are slightly inferior to the MA-cGAN model in terms of stability and maximum values, and exhibit certain deficiencies.

### Qualitative results

**Fig 7:**
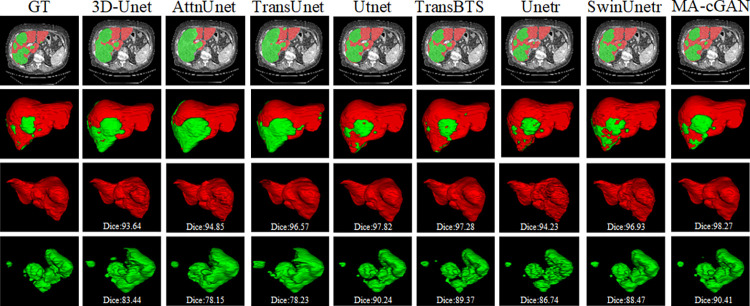
Visual comparisons of the segmentation results were performed on the LiTS dataset with other methods. MA-cGAN demonstrated remarkable performance in both tumor and liver segmentation tasks, particularly excelling in handling small targets and irregular regions, thereby exhibiting more accurate segmentation performance.

To further validate the effectiveness of the MA-cGAN network in liver and tumor segmentation tasks, we visualized the segmentation results on the LiTS dataset. The tumor models in the dataset contain both large target regions and small, blurry regions with irregular surfaces, which pose high demands on the model’s ability to segment multi-scale targets. By visually comparing the segmentation results, we can better evaluate the performance of each model in liver and tumor segmentation tasks. The comparisons are shown in Fig 7.

The comparative results reveal that the liver models segmented by 3D-Unet [34] and AttnUnet [11] exhibit a relatively rough surface, and there are more false positive regions in the tumor models. In contrast, the liver segmentation results obtained by MA-cGAN exhibit a smoother surface, fewer false positive regions in the tumor models, and finer edges, which are closer to the real samples. On the other hand, networks such as UTNet [18], TransBTS [19] and Unetr [20], achieve good segmentation performance, but the introduction of Transformer modules significantly increases the number of parameters and computational requirements, demanding more computational resources. MA-cGAN, on the other hand, demonstrates excellent performance while utilizing fewer parameters and computational resources, thereby proving its suitability for medical-assisted treatments.

**Fig 8:**
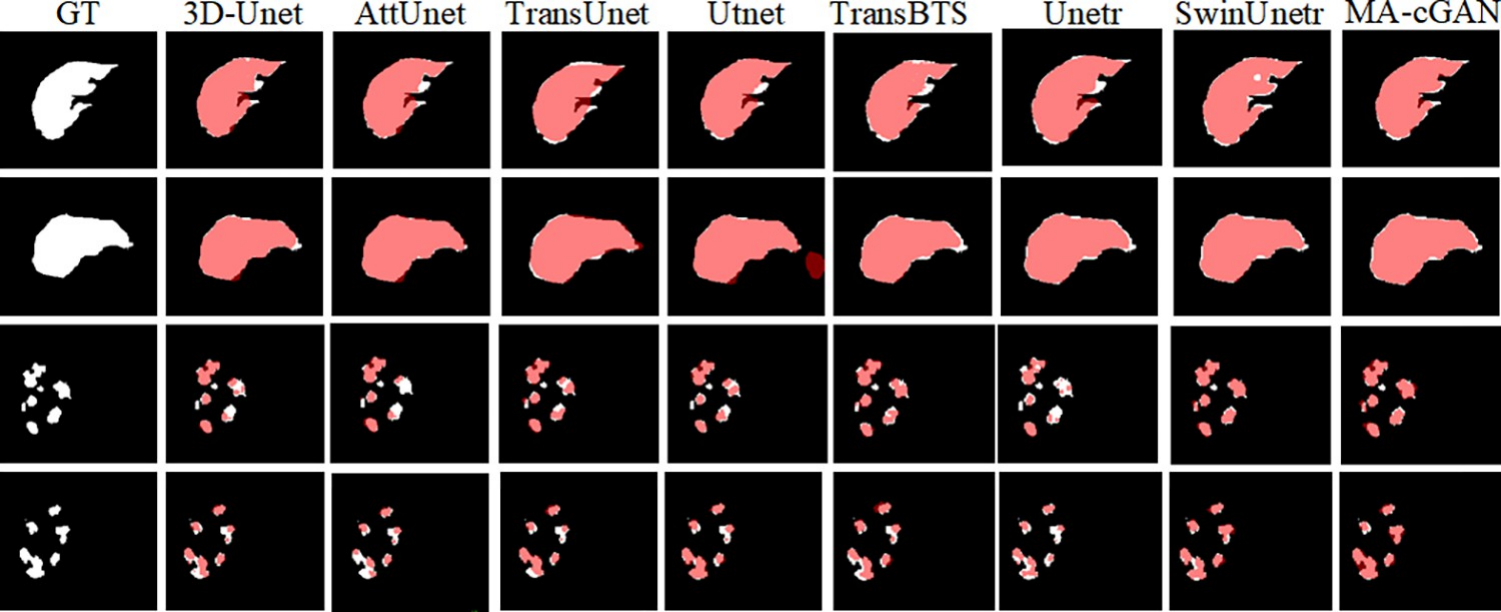
Visual comparison of 2D section. Compared to other networks. The segmentation results of MA-cGAN contain fewer false positive and false negative regions, resulting in higher segmentation accuracy.

We further compared the segmentation results using two-dimensional cross-sectional views to better observe any false positive or false negative regions present in the segmentation results. The comparative results are shown in Fig 8.

Fig 8 illustrates the comparative results of our proposed MA-cGAN model against several common models for liver and tumor segmentation tasks. The first two rows depict liver segmentation results, while the last two rows show tumor segmentation results. The white areas in the figure represent the Ground Truth (GT) labels, and the red areas denote the segmentation results of each model.

In liver segmentation, the MA-cGAN model exhibits results closest to the GT, demonstrating its superior performance in accurately segmenting the liver. In contrast, other models show varying degrees of error in their segmentation results. For instance, 3D-Unet and AttUnet models exhibit noticeable false negative regions along the liver edges (white areas not covered by red), indicating inaccuracies in certain edge recognition scenarios. Although TransUnet, UTNet, and TransBTS models approximate the overall shape of GT, they still exhibit some false positive areas (red areas extending beyond white), suggesting potential over-segmentation in specific cases. Unetr and SwinUnetr models show significant errors in certain regions, particularly along the liver edges. The MA-cGAN model excels in liver segmentation by accurately capturing both the edges and detailed parts of the liver, significantly reducing false positive and false negative regions.

In tumor segmentation, the MA-cGAN model similarly performs exceptionally well, with segmentation results closely matching GT, accurately identifying tumor shapes and positions. Conversely, other models show considerable discrepancies in tumor segmentation tasks. 3D-Unet and AttUnet models display numerous false negative areas in tumor segmentation, especially along tumor edges, indicating challenges in accurate tumor recognition. While TransUnet and UTNet models achieve closer results to GT, they still exhibit false positive areas in certain regions, indicating potential over-segmentation in specific scenarios. TransBTS, Unetr, and SwinUnetr models demonstrate evident false positive and false negative occurrences in specific areas. The MA-cGAN model excels in tumor segmentation by accurately identifying both the edges and detailed parts of tumors, significantly reducing false positive and false negative regions.

In summary, the MA-cGAN model demonstrates outstanding performance in both liver and tumor segmentation tasks, accurately capturing the edges and detailed parts of the target regions while significantly reducing false positive and false negative regions. This validates the superiority of our proposed network for these two tasks. While other models also perform well, they exhibit slightly inferior stability and accuracy compared to the MA-cGAN model, with some shortcomings.

### Ablation

#### Effect of the hyperparameters in Tversky loss

The sizes of *α* and *β* values in Tversky Loss have a certain impact on the segmentation performance of the MA-cGAN network. The values of these two parameters are typically in the range of 0, 1, and the sum of a and b is equal to 1. In this study, we conducted ablation experiments to investigate the effects of different α and β values on the performance of the MA-cGAN network. The experimental results are shown in Table 2.

**Table 2:** The impact of *α* and *β* on the performance of MA-cGAN.

*α*	*β*	Liver				Tumor			
		Dice	HD95	ASD	Jaccard	Dice	HD95	ASD	Jaccard
0.5	0.5	96.53	2.98	0.68	93.57	76.25	14.06	2.45	59.71
0.6	0.4	96.82	2.78	0.73	94.34	77.84	12.10	2.25	64.65
0.7	0.3	**96.95**	**2.15**	0.54	**94.54**	**78.53**	**12.12**	**2.01**	**65.24**
0.8	0.2	96.64	2.46	0.64	93.81	78.48	14.53	2.42	61.48
0.9	0.1	96.72	2.35	**0.53**	92.68	76.95	13.85	2.33	64.87

If *α* is too large, the model will prioritize accuracy over class balance, and it may tend to predict all regions as healthy tissues to maximize accuracy. However, this can result in a very low recall, potentially missing some true tumor lesions and leading to false negatives. These lesions may require treatment in the early stages, and missing the optimal treatment window could worsen the condition and even jeopardize the patient’s life. Additionally, this can cause a performance drop when dealing with minority classes, leading to a decrease in overall image segmentation performance.

On the contrary, if *β* is too large, it can lead to false positives. Although this may result in some unnecessary treatments, the impact is relatively smaller since these erroneous judgments can be corrected through further examination and diagnosis.

In medical prediction tasks, false negatives may pose greater risks and harm to patients compared to false positives. After conducting multiple ablation experiments, this study sets the value of *α* as 0.7 and *β* as 0.3 to achieve a balanced trade-off between precision and recall, adapting to the liver and tumor segmentation tasks.

### Effect of the cGAN and MA module

To further validate the effectiveness of the proposed Conditional Generative Adversarial Network (cGAN) and Multi-Axis Attention (MA) mechanism, we used the U-Net segmentation network mentioned in the text as a baseline. We incorporated cGAN and MA separately into the baseline, and also conducted an ablation experiment with MA-cGAN to evaluate the performance improvement of cGAN and MA on liver and tumor segmentation. The quantitative segmentation results of the experiments are compared and presented in Table 3.

**Table 3:** Quantitative segmentation results of cGAN and MA modules.

Model	Liver				Tumor				Params	Flops
	Dice	HD95	ASD	Jaccard	Dice	HD95	ASD	Jaccard		
Baseline	94.65	6.51	1.21	90.81	60.89	32.68	6.87	48.25	2.90M	36.34G
Baseline+cGan	95.20	6.67	1.22	91.15	66.71	23.02	6.47	55.03	3.74M	38.41G
Baseline+MA	96.57	2.54	0.62	**94.68**	72.52	14.89	4.93	57.67	4.15M	39.86G
MA-cGAN	**96.95**	**2.15**	**0.54**	94.54	**78.53**	**12.12**	**2.01**	**65.24**	4.99M	41.93G

**Fig 9:**
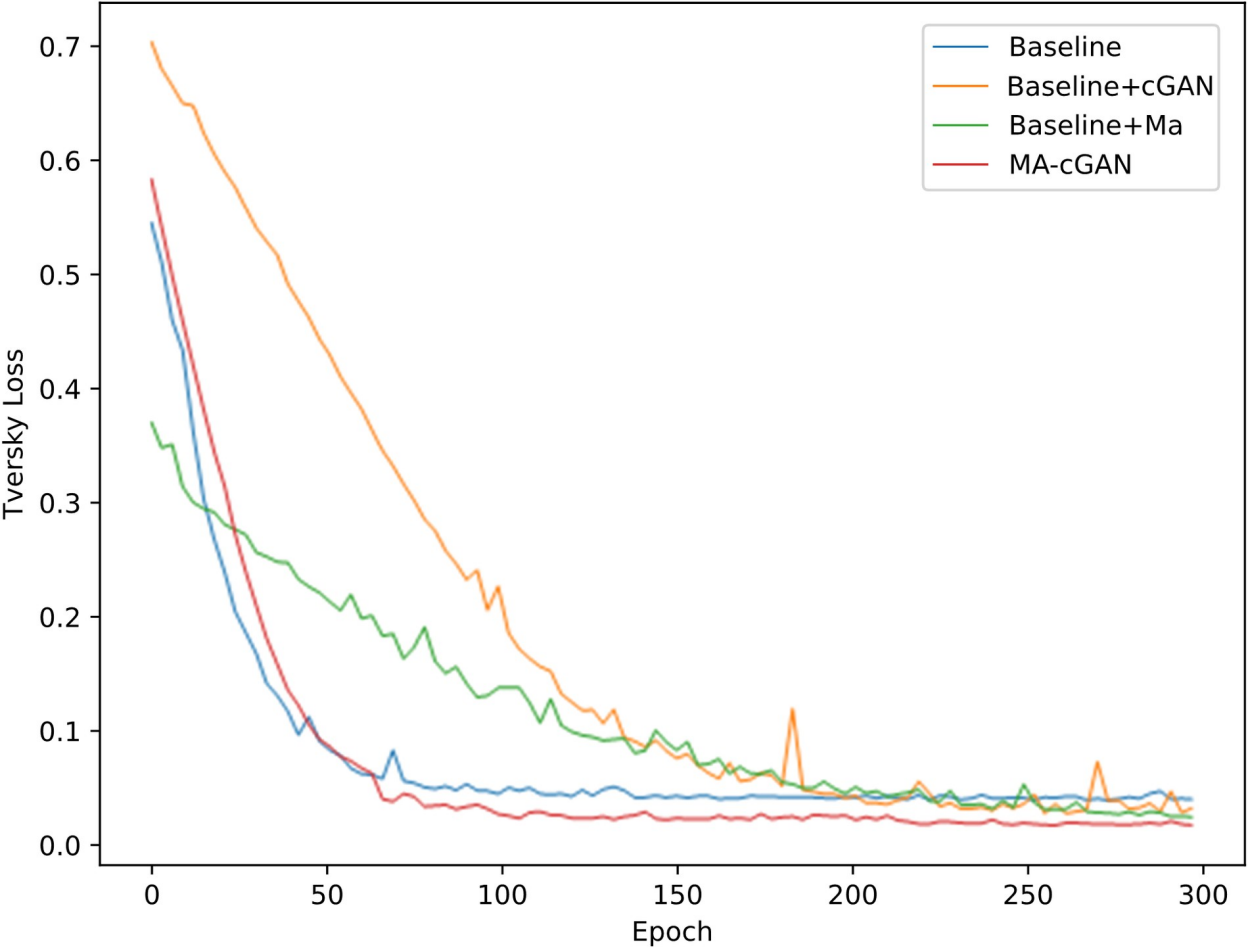
Comparison of validation set Tversky loss during training with cGAN and MA Modules in the Baseline. The downward trend of the Tversky Loss can be used to compare the segmentation accuracy and convergence speed of the network.

After each training epoch, we also computed the Tversky Loss between the segmentation results on the validation set and the corresponding ground truth labels. summed these values, and took their average. The trend of average Tversky Loss on the validation set during the training process is shown in Fig 9.

It can be observed that although the baseline exhibits a rapid decrease in Tversky Loss during training, its performance capacity is limited, as it fails to adequately capture the data distribution and patterns. This limitation can result in the model’s inability to accurately learn the features of real data during the training process. Therefore, even after training, the baseline still exhibits relatively high Tversky Loss.

After introducing the cGAN module, the adversarial training between the generator and discriminator allows the generator to learn more intricate liver and tumor image features, resulting in more accurate segmentation boundaries and improved segmentation accuracy. However, this adversarial training process requires more iterations and training time to converge. Therefore, during the training process, the Tversky Loss of the cGAN model decreases at a slower rate compared to the Baseline model.

After incorporating the MA module into the baseline, MA can extract two-dimensional projection feature information along three axes (X, Y, Z) and compress and integrate the multi-axis information, thereby reducing the dimensionality of the features and enhancing the focus on key feature information. Furthermore, by adaptively adjusting the weights between different axes, MA can better capture the correlations between different axes and modalities, further improving the accuracy of liver and tumor segmentation.

**Fig 10:**
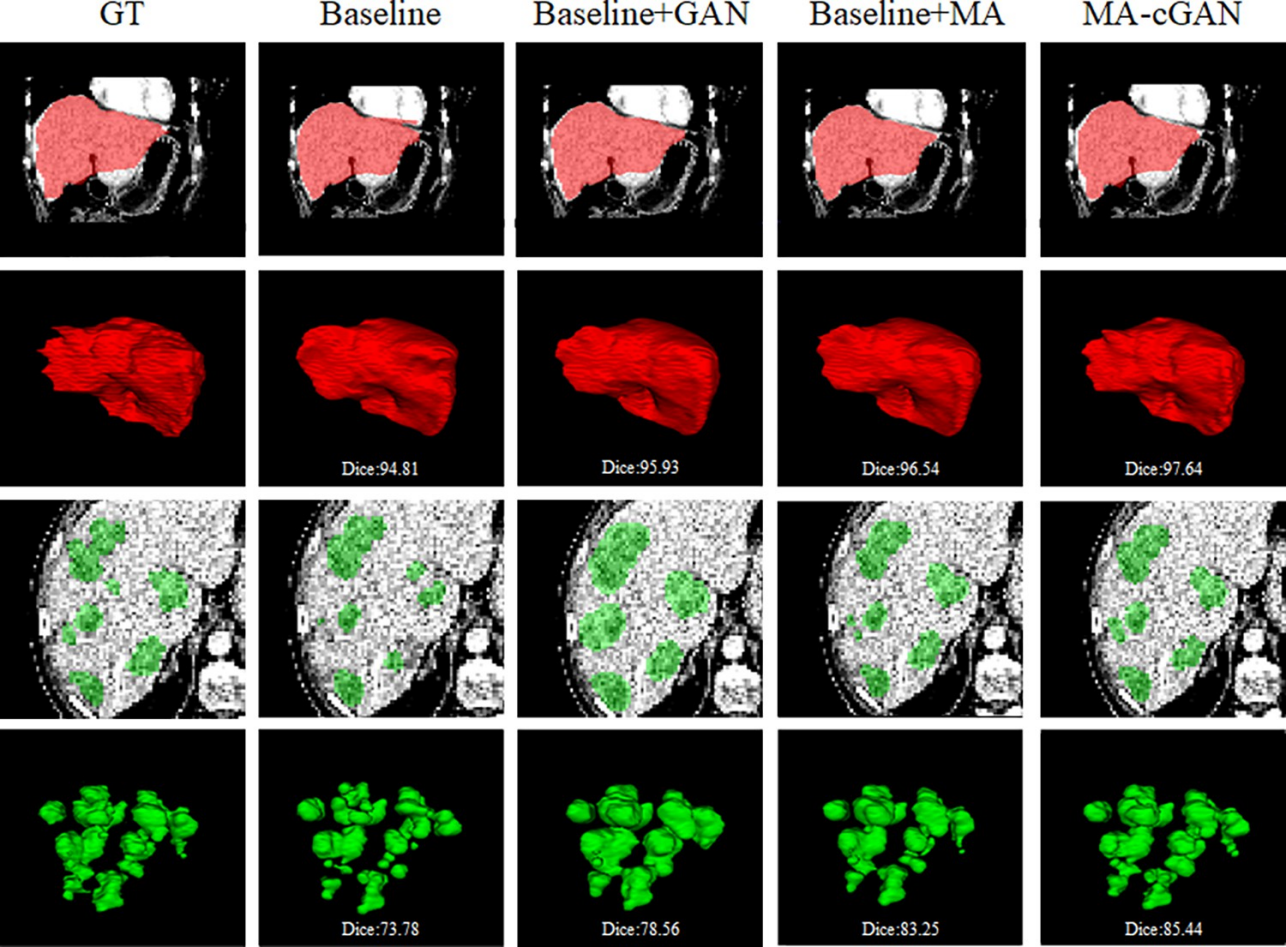
Visualization comparison of segmentation results from GAN and MA ablation experiments. Compared to the Baseline, the inclusion of GAN and MA both improved the segmentation performance of the network, leading to higher accuracy in the segmentation results for both liver and tumor.

GAN and MA have improved the segmentation performance of the network for the liver and tumor. The visual comparison of the segmentation results is shown in Fig 10.

Compared to the ground truth labels, the segmentation results of the Baseline model only roughly preserve the segmentation shapes, and the accuracy in capturing edge details is relatively low. After incorporating GAN, the generator can generate images that are similar to the real liver segmentation results, closer to the actual liver shape and structure. The discriminator network of GAN provides feedback on the differences between the generated results and the real results, aiding the generator in learning more accurate liver boundaries and tumor regions. With the addition of MA, the network can capture multi-axis feature information for fusion, resulting in a greater focus on important regions within the liver or regions with pathologies, leading to higher segmentation accuracy in these areas. MA-cGAN combines GAN and MA, retaining the high similarity of GAN and the high attention of MA, resulting in liver and tumor segmentation results that are closer to the ground truth, with clearer edge details and improved segmentation performance.

## Conclusions

In the current diagnosis of liver diseases, accurately segmenting the liver and tumor models from CT images is crucial as it can assist doctors in further treatment planning. In this paper, we propose the MA-cGAN network, which incorporates the MA mechanism to fuse feature information from three directions and integrates it into a U-shaped segmentation network as the generator. Together with the discriminator, they form a conditional generative adversarial network, where adversarial training is conducted in an alternating manner to enhance the segmentation accuracy of the generator. In the results on the LiTS test dataset, MA-cGAN demonstrates segmentation results that are closer to the true data distribution compared to other networks, even with smaller parameters and limited data. This makes it suitable for 3D printing and preoperative planning of liver tumor models.

In existing medical segmentation, there is a challenge of limited datasets and insufficient labeled data. To further improve the automatic segmentation network, the following improvements will be made based on the following ideas: utilizing the generator to generate high-quality predicted labels from unlabeled samples, and incorporating these predicted labels together with the ground truth labels as training data to achieve semi-supervised training. This approach aims to address the scarcity of labeled samples.
